# An Analysis of Music Perception Skills on Crowdsourcing Platforms

**DOI:** 10.3389/frai.2022.828733

**Published:** 2022-06-14

**Authors:** Ioannis Petros Samiotis, Sihang Qiu, Christoph Lofi, Jie Yang, Ujwal Gadiraju, Alessandro Bozzon

**Affiliations:** ^1^Department of Software Technology, Delft University of Technology, Delft, Netherlands; ^2^Hunan Institute of Advanced Technology, Changsha, China

**Keywords:** human computation, music annotation, perceptual skills, music sophistication, knowledge crowdsourcing

## Abstract

Music content annotation campaigns are common on paid crowdsourcing platforms. Crowd workers are expected to annotate complex music artifacts, a task often demanding specialized skills and expertise, thus selecting the right participants is crucial for campaign success. However, there is a general lack of deeper understanding of the distribution of musical skills, and especially auditory perception skills, in the worker population. To address this knowledge gap, we conducted a user study (*N* = 200) on Prolific and Amazon Mechanical Turk. We asked crowd workers to indicate their musical sophistication through a questionnaire and assessed their music perception skills through an audio-based skill test. The goal of this work is to better understand the extent to which crowd workers possess higher perceptions skills, beyond their own musical education level and self reported abilities. Our study shows that untrained crowd workers can possess high perception skills on the music elements of *melody, tuning, accent*, and *tempo*; skills that can be useful in a plethora of annotation tasks in the music domain.

## 1. Introduction

Several studies have shown the ability of crowd workers to successfully contribute to the analysis and annotation of multimedia content, both based on simple perceptual skill, e.g., for image analysis (Sorokin and Forsyth, 2008) and domain-specific knowledge, (Oosterman et al., 2015). Musical content is no exception, and research has shown that the general crowd can be successfully involved in the annotation (Samiotis et al., 2020) and evaluation (Urbano et al., 2010) processes of music-related data and methods. Plenty of music annotation tasks, (Lee, 2010; Mandel et al., 2010; Speck et al., 2011; Lee and Hu, 2012; Lee et al., 2012) can be routinely found on microtask crowdsourcing platforms, mostly focused on descriptive (Law et al., 2007) and emotional (Lee, 2010) tagging.

Music, as a form of art, often requires a multifaceted set of skills to perform and certain expertise to analyse its artifacts. There are cases that require advanced music perceptual skills (such as the ability to perceive changes in melody) and music-specific knowledge. However, both in literature and in practice, it is rare to encounter such crowdsourcing tasks. Consider, for example, annotation tasks targeting classical music, e.g., music transcription, performance evaluation, or performance annotation. Classical music is a genre featuring artworks with high musical complexity; it is no surprise that corresponding analysis and annotation tasks are often exclusively performed by musical experts and scholars. This unfortunately hampers current efforts to digitize and open up classical music archives, as scholars and experts are expensive and not easily available. Here, the ability to utilize microtask crowdsourcing as an annotation and analysis approach could bring obvious advantages. But how likely it is to find advanced music-related perceptual skills on crowdsourcing platforms? With the goal of answering this broad research question, in this paper we scope our investigation on the following two aspects:

- **[RQ1]** How are different music perception skills and self-reported music-related knowledge distributed among crowd workers of different platforms?
- **[RQ2]** How are music perception skills associated to domain and demographic attributes?

Studies on human cognition and psychology have shown that people can possess innate music perception skills without previous formal training (Ullén et al., 2014; Mankel and Bidelman, 2018). However, the majority of those studies have been conducted in labs, under controlled conditions and with limited amounts of participants.

In our work, we set out to measure the music sophistication and perception skills of crowd workers operating on the Prolific^1^ and Amazon Mechanical Turk^2^ crowdsourcing platforms. We chose to conduct our study on these two different platforms, in order to diversify our participant pool and identify potential differences between them. In its present form, this study expands the preliminary study as presented in Samiotis et al. (2021), by diversifying the participant pool and complementing the analysis with additional methods.

We designed a rigorous study that employs validated tools to measure the musical sophistication of the users and quantify their music perception skills: the Goldsmith's Music Sophistication Index (GMSI) questionnaire (Müllensiefen et al., 2014) and the Profile of Music Perception Skills (PROMS) active skill test (Law and Zentner, 2012), respectively (and more specifically its shorten version: Mini-PROMS). These tools allow for a general overview of musical ability characteristics, but also a more detailed understanding through their subcategories (e.g., musical training and melody perception skills). By juxtaposing passive methods of assessment (questionnaire) with the active evaluation of auditory skills, we aim to gather a better understanding of workers' actual skills on musical aspects, beyond their subjective self-assessment. With GMSI, we are able to evaluate a person's ability to engage with music through a series of questions focusing on different musical aspects. PROMS on the other hand, allows for a more objective way to measure a person's auditory music perception skills (e.g., melody, tuning, accent, and tempo perception) through a series of audio comparison tests. To the best of our knowledge, this is the first attempt to use PROMS in an online crowdsourcing environment and the measured perception skills can offer valuable insights to the auditory capabilities of the crowd.

Our findings indicate that pre-existing musical training is not common among crowd workers, and that music sophistication aspects are not necessarily predictive of actual music perception skills. Instead, we observe that the majority of workers show an affinity with specific sets of skills (e.g., we found a surprising number of *musical sleepers* — workers without formal training but still high music perception skill test results). As a whole, our study paves the way for further work in worker modeling and task assignment, to allow a wider and more refined set of microtask crowdsourcing tasks in the domain of music analysis and annotation.

## 2. Related Work

There is a long history of studies on perception and processing of music by humans; from the analysis of the socio-cultural variables influencing a person's musicality amplitude (Hannon and Trainor, 2007), to the study of musicality from a genetics' base (Gingras et al., 2015). In all cases, inherent music processing capabilities have been found in people and they seem to be connected with basic cognitive and neural processes of language since early stages of development (Liberman and Mattingly, 1985; Koelsch et al., 2009). Even people with *amusia*, a rare phenomenon where a person can't distinguish tonal differences between sounds (Peretz and Hyde, 2003), they can still process and replicate rhythm correctly (Hyde and Peretz, 2004).

In Müllensiefen et al. (2014), we find a large scale study on musical sophistication through the use of the GMSI survey, on a unique sample of 147,663 people. GMSI is particularly calibrated to identify musicality in adults with varying levels of formal training. It is targeted toward the general public, and can prove less effective to distinguish fine differences between highly trained individuals. Musical sophistication in the context of that study, and ours, encompasses musical behaviors and practices that go beyond formal training on music theory and instrument performance. Their findings show that musical sophistication, melody memory and musical beat perception are related. The survey has been translated and replicated successfully (on smaller samples) in French (Degrave and Dedonder, 2019), Portuguese (Lima et al., 2020), Mandarine (Lin et al., 2021), and German (Schaal et al., 2014).

Our study draws connections to those findings and aims to shed light into the musical capabilities of people on crowdsourcing platforms. The demographics and conditions of the studies presented so far cannot be easily compared to those of online markets. Users on those platforms are participating in such studies through monetary incentives, and the conditions (equipment, location, potential distractions, etc.) under which they perform the tasks cannot be controlled as in a lab environment, as indicated in Totterdell and Niven (2014), Gadiraju et al. (2017), and Zhuang and Gadiraju (2019).

Currently, crowdsourced music annotation is primarily utilized for descriptive (Law et al., 2007) and emotional (Lee, 2010) tagging. Large-scale music data creation and annotation projects such as Last.fm^3^ and Musicbrainz^4^, are largely depended on human annotation, but from users of their respective online social platforms. A survey on the applicability of music perception experiments on Amazon Mechanical Turk (Oh and Wang, 2012), showed that online crowdsourcing platforms have been underused in the music domain and the status has not changed radically since then. Through our study, we want to examine the capabilities of the crowd on processing music audio and showcase their capabilities, in an attempt to encourage further research and utilization of crowdsourcing in the music domain. Although our focus on audio perception separates our work from visual-based studies on music perception, it is meaningful to mention that visualization techniques for music tasks have proven effective for certain use cases such as music plagiarism detection in De Prisco et al. (2016) and De Prisco et al. (2017) but also harmonic structure perception in music, in Malandrino et al. (2015).

## 3. Experimental Design

The main focus of this study is to offer insights into the musical characteristics and perception skills of workers operating on crowdsourcing platforms. We therefore designed our experiment to capture these attributes through methods that can be used online, and that do not require pre-existing musical knowledge. We used two methods: 1) the *GMSI* questionnaire to evaluate the *musical sophistication* (musical training, active engagement and other related musical characteristics) (Müllensiefen et al., 2014) of workers and 2) the *Mini-PROMS* test battery to evaluate their auditory music perception skills. We then compare the obtained results, paying specific attention to the overlapping aspects of musical sophistication and music perception skills. With this experiment, we are also interested in identifying “*musical sleepers*” and “*sleeping musicians*”, a notion originally presented in Law and Zentner (2012). A musical sleeper is a person with little to no musical training but with high performance in the perception test, while a sleeping musician indicates the opposite.

### 3.1. Procedure

**Figure 1:**
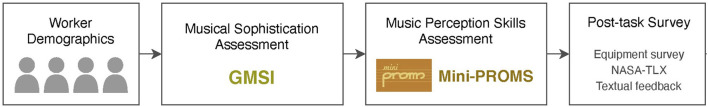
The four steps in the music perception skills study.

After a preliminary step where workers are asked basic demographic information (age, education, and occupation), the study is composed of four consecutive steps (Figure 1), each devoted to collecting information about specific attributes corresponding to the crowd workers: (1) Musical Sophistication Assessment (*GSMI*), (2) Active Music Perception Skill Assessment (*Mini-PROMS*) and (3) Post-task Survey collecting information on workers audio-related conditions, and perceived cognitive load.

### 3.2. Questionnaires and Measures

#### 3.2.1. Capturing Musical Sophistication of Workers

Musical behaviors of people such as listening to music, practicing an instrument, singing or investing on vinyl collections, all show the affinity of a person toward music. The degree to which a person is engaged to music through these behaviors, constitutes the musical sophistication. Musical sophistication can be measured as a psychometric construct through the *GMSI* questionnaire, which collects self-reported musicality through emotional responses, engagement with music, formal training, singing capabilities and self-assessed perception skills. It is an instrument specifically designed to capture the sophistication of musical behaviors, in contrast to other questionnaires such as Musical Engagement Questionnaire (MEQ) (Werner et al., 2006), which measures the spectrum of psychological facets of musical experiences. More specifically, the musical sophistication of people based on Müllensiefen et al. (2014), is organized into the following five facets:

- Active Engagement: this aspect determines the degree to which a person engages with music, by listening to and allocating their time/budget to it;
- Perceptual Abilities: this aspect assesses the skill of perceiving (mainly auditory) elements of music. This is an important subscale in our study, since the self-assessed perceptual skills of the workers in GMSI can be directly compared to those we actively measure in Mini-PROMS;
- Musical Training: this aspect reports the years of training on aspects of music (e.g., theory, performing an instrument), which can indicate the formal expertise that a person has in the domain;
- Emotions: this aspect determines the emotional impact of music on that person;
- Singing Abilities: this aspect evaluates the ability to follow along melodies and tempo (beat) of songs.

GMSI offers additional questions outside the subscales, which capture specific properties of the participant: 1) “Best Instrument”, which represents which instrument the user knows to play the best, 2) “Start Age”, which age the participant starting learning an instrument and 3) “Absolute Pitch”, which indicates if the person can understand correctly the exact notes of a sound frequency. Absolute pitch is a very rare trait that develops during the early stages of auditory processing (Burkhard et al., 2019) but can deteriorate through the years (Baharloo et al., 1998). As such, a person with perfect pitch perception, could have an advantage on a melody perception test, thus we included it with the rest of the subscales.

The original GMSI questionnaire contains 38 main items and 3 special questions, and considering the rest of the study's parts, we chose to reduce its size while keeping its psychometric reliability. For that purpose, we consulted the GSMI online “configurator”^5^ which allows to select the number of items per subscales and estimates the reliability of the resulting questionnaire based on the questions it selects. We reduced the size of the questionnaire to 34 questions, and preserved the special question about “Absolute Pitch”, resulting in 35 questions in total.

In the GMSI questionnaire each question from the subscales uses the seven-point Likert scale (Joshi et al., 2015) for the user's responses, with most questions having “Completely Agree”, “Strongly Agree”, “Agree”, “Neither Agree Nor Disagree”, “Disagree”, “Strongly Disagree” and “Completely Disagree” as options. Few questions offer numerical options for topics (e.g., indicating the time spent actively listening to music, or practicing an instrument). The workers are not aware of the subscale each question belongs to. The index of each subscale of GMSI is calculated with the aggregated results of the relevant questions. The overall index of “General Music Sophistication” is calculated based on 18 questions out of the total 34 items of the subscales; these 18 questions are predefined by the designers of the questionnaire; the question about “Absolute Pitch” does not contribute to the total index.

Using the GMSI questionnaire is close to the typical methods used to assess the knowledge background of annotators in other domains. Especially the questions of “Musical Training” follow standard patterns to assess the formal training of a person in a domain, thus a certain objectivity can be expected (assuming good faith from the workers). However, the rest of the categories are based purely on subjective indicators and self-reported competence, which can potentially misrepresent the true music behaviors and capabilities of a worker. For this reason, it is necessary to understand the best practices that could reliably predict a worker's performance to a music annotation task. To that end, we compare the workers' input in such questionnaires, and specifically on GMSI, to the music perceptual skills they might possess, which we measure through an audio-based, music perception skill-test.

#### 3.2.2. Measuring Music Perception Skills of Workers

The music perception skill test is based on the well-establish *Profile of Music Perception Skills* (PROMS) test (Law and Zentner, 2012). Its original version is quite extensive and its completion can take more than an hour, as it covers several music cognition aspects like Loudness, Standard rhythm, Rhythm-to-melody, Timbre, Pitch and more. Considering the possibly low familiarity of crowd workers with these tasks and its inherent difficulty, we opted for a shorter version, the *Mini-PROMS* (Zentner and Strauss, 2017), which has also been adopted and validate in the context of online, uncontrolled studies.

Mini-PROMS is a much shorter battery of tests ( 15 min completion time), which still covers the “Sequential” and “Sensory” subtests. It can measure a person's music perception skills, by testing their capability to indicate differences on the following musical features:

- Melody: A sequence of notes, with varying density and atonality
- Accent: The emphasis of certain notes in a rhythmic pattern
- Tuning: The certain frequency of notes, when played in a chord
- Tempo: The speed of a rhythmic pattern.

The musical aspects selected in this test are argued to well represent the overall music perception skills of a person, only in a more concise way. This version retains test–retest reliability and internal consistency values close to the original PROMS test (Law and Zentner, 2012), validating it for our research purposes. Note that, although reduced in size, these four skills are required to enable a broad range of music-related research, such as beat tracking, tonal description, performance assessment and more.

For each of the 4 musical aspects workers receive a brief explanation and an example case to familiarize the user with the test. Each test after the introduction presents a reference audio sample twice and a comparison sample once. The two audio samples can differ based on the musical aspect tested and the worker is asked if the samples are indeed same or differ. The authors of PROMS have put particular effort on distinguishing the musical aspects from each other, to make the skill evaluation as close as possible to the musical aspect tested. Finally, to minimize cognitive biases due to enculturation (Demorest et al., 2008) the audio samples have been created using less popular instrument sounds, such as harpsichord and “rim shots”. Meanwhile, the structure of audio samples and the aspect separation allow for a more precise measurement of a person's perception skill.

The categories of “Melody” and “Accent” have 10 comparisons each, while “Tuning” and “Tempo” have 8. After the user has listened to the audio samples, they are asked to select between “Definitely Same”, “Probably Same”, “I don't know”, “Probably Different”, and “Definitely Different”. The participant is then rewarded with 1 point for the high-confidence correct answer, while the low-confidence one rewards 0.5 point. The subscale scores are calculated through a sum of all items within the scale and divided by 2. The total score is an aggregated result of all subscale scores. During the test, the user is fully aware of the subscale they are tested for, but the name of “Tempo” is presented as “Speed” (original creators' design choice).

#### 3.2.3. Self-Assessment on Music Perception Skills

Self-assessment can often misrepresent an individual's real abilities (Kruger and Dunning, 1999). For that reason, we employed a survey to study this effect its manifestation with music-related skills. After Mini-PROMS test, the worker has to input how many of the comparisons per subscale they believe they correctly completed—this information is not known to them after executing the Mini-PROMS test. Therefore, they are presented with 4 questions, where they have to indicate between 0 and the total number of tests per subscale (10 for “Melody”/“Accent” and 8 for “Tuning”/“Tempo”). Finally, the results of this survey are compared to the score of workers on the “Perceptual Abilities” subscale of GMSI, which also relies on self-assessment. We expect workers to re-evaluate their own skills, once exposed to the perception skill test.

#### 3.2.4. Post-task Survey

As a final step of the task, the worker is presented with three post-task surveys: (1) a survey on the audio equipment and the noise levels around them, (2) a survey on the cognitive load they perceived and (3) an open-ended feedback form.

The audio equipment survey consisted of four main questions, to retrieve the type of equipment, its condition and the levels of noise around them during the audio tests. Insights on these can help us understand the to what extent the equipment/noise conditions affected Mini-Proms test, which is audio-based. More specifically, we asked the following questions:

1. What audio equipment were you using during the music skill test?
2. What was the condition of your audio equipment?
3. Does your audio equipment have any impairment?
4. How noisy was the environment around you?

The options regarding the audio equipment were: “Headphones”, “Earphones”, “Laptop Speakers”, and “Dedicated Speakers”. For the condition questions (2) and (3), we used the unipolar discrete five-grade scales introduced in ITU-R BS (2003), to subjectively assess the sound quality of the participants' equipment. Finally, for question (4) on noise levels, we used the loudness subjective rating scale, introduced in Beach et al. (2012).

In the second part of post-task survey, the workers had to indicate their cognitive task load, through the NASA's Task Load IndeX (NASA-TLX) survey^6^. The survey contains six dimensions—Mental Demand, Physical Demand, Temporal Demand, Performance, Effort, and Frustration. Workers use a slider (ranging from 0 to 20, and later scaled to 0 to 100) to report their feelings for each of the six dimensions. A low TLX score represents the music skill test is not mentally, physically, and temporally demanding, and it also indicates less effort, and less frustration perceived by the worker, while completing the entire study.

Finally, we introduced an free-form textual feedback page, where users were encouraged to leave any comments, remarks, or suggestions for our study.

### 3.3. Worker Interface

The worker interfaces of our study is using VueJS^7^, a JavaScript framework. The first page of our study contained general instructions for the study alongside estimated completion times for each part of it. Each page thereafter, contained an interface for each of the steps in our study, as seen in Figure 1.

**Figure 2:**
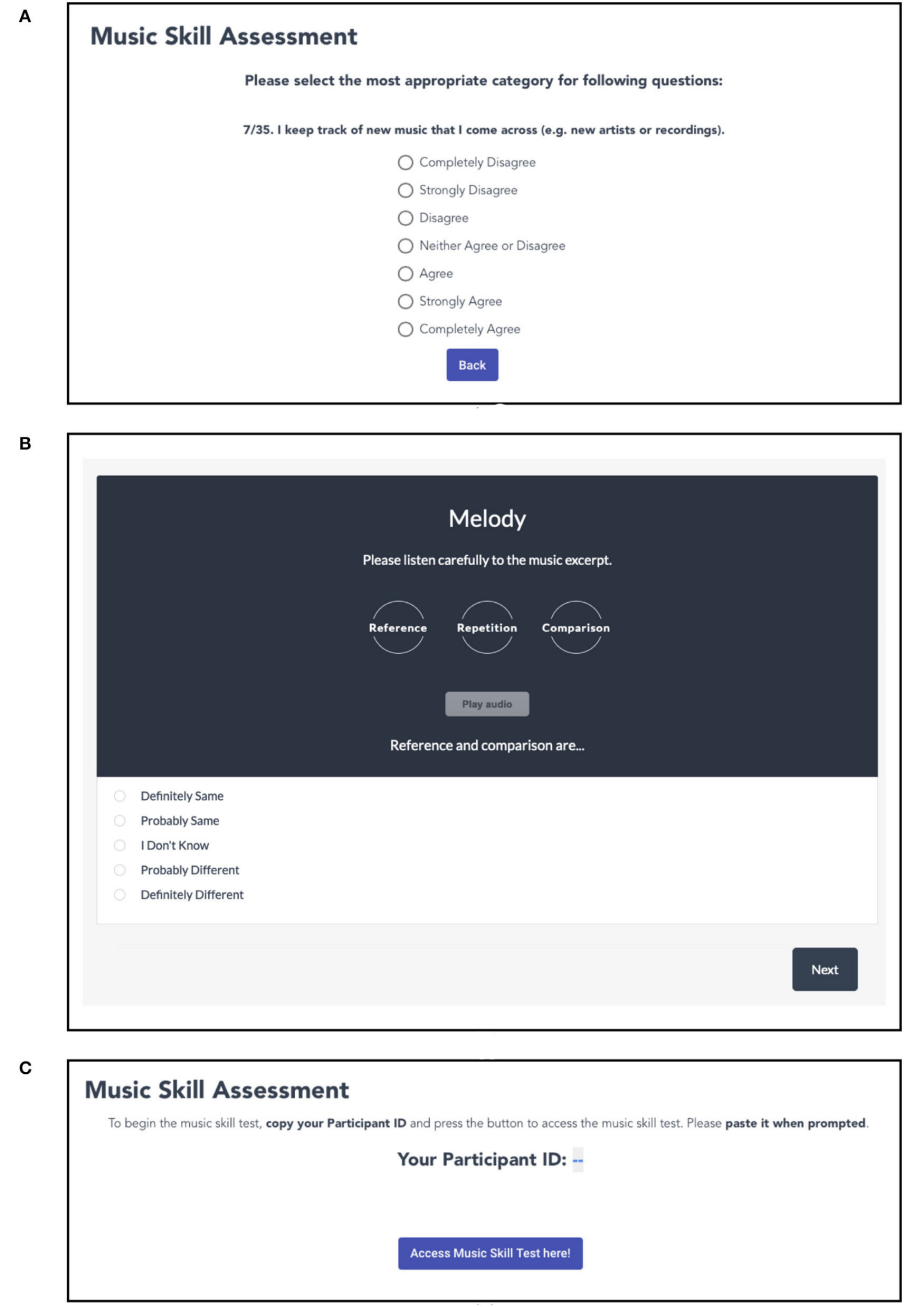
Interfaces of the study (**A**, GMSI questionnaire, **B**, Mini-PROMS, and **C**, Participant ID prompt).

To assist navigation through the GMSI questionnaire, we implemented the questionnaire interface to show one question at a time. We added a small drifting animation to show the next question, when they select their answer in the previous one. We also added a “back” button, in case they wanted to return to a previous question and alter their answer. They could track their progress through the questionnaire from an indication of the number of the question and the total number of questions (see Figure 2).

While we retrieved the questions for GMSI and implemented them in our study's codebase, for PROMS we wanted to use the exact conditions and audio-samples as in Zentner and Strauss (2017). To replicate their test faithfully, the creators of PROMS (Law and Zentner, 2012) kindly gave us access to their Mini-PROMS interfaces (example interface in Figure 2). Mini-PROMS is implemented on LimeSurvey^8^ and users were redirected to it after the completion of GMSI.

After the GMSI questionnaire, workers were introduced to the page seen in Figure 2. There, they had to copy their Participant ID (retrieved programmatically from the crowdsourcing platforms) and use it in the Mini-PROMS interface later, so we could link their test performance (stored in LimeSurvey), with their entries in our database. At the end of Mini-PROMS, the users were redirected back to our study through a provided URL.

In the final stage of our study, the participants were greeted and provided a “completion code”, which they could submit on back on their respective platform, to complete the task.

### 3.4. Participants, Quality Control, and Rewards

On Prolific, we recruited 100 crowd workers to complete our study. We applied a participant selection rule for “Language Fluency”: English, as all of our interfaces were implemented in English. Only crowd workers whose overall approval rates were higher than 90% could preview and perform our study. On Amazong Mechanical Turk, we recruited 100 crowd workers as well, where we set their approval rate to “greater than 90%”.

To assess the quality of the user input, we included attention check questions on the GMSI and NASA-TLX interfaces of the study. More specifically, we included three attention check questions in GMSI, asking the participants to select a specific item in the same seven-point Likert scale. In the NASA-TLX survey, we included a question asking the users to select a specific value out of the 21 available in the scale of the survey.

We set the reward on Prolific and Amazon Mechanical Turk for completing our study to 3.75 GBP (5.2 USD). Upon the completion of our study on both platforms, workers immediately received the reward. The average execution time was 32.5 min, resulting in the hourly wage of 7.5 GBP (10.3 USD), rated as a “good” pay by the platforms.

## 4. Results

While investigating the data we gathered in our study, we followed similar analysis steps for both platforms. The data were first cleaned up based on our attention check questions and we only kept demographic data that we had actively asked the participants (dropped platform-based demographics).

We proceeded with identifying the distribution characteristics of each variable from the different parts of our study (GMSI and Mini-PROMS subcategories, NASA-TLX and equipment questions). Combined with the intercorrelations per study part, we gained important insights on the attributes of each variable and their relations. These results are compared to those of the original GMSI and Mini-PROMS studies, to assess the differences between the different participants' pools. Finally, we run a Multiple Linear Regression, to assess which factors seem to be the best predictors for the music perception skills of a crowd worker (e.g., musical training, equipment quality etc.).

### 4.1. Prolific

Of the 100 workers recruited from Prolific, 8 of them failed at least one attention check question(s); 5 of them provided invalid/none inputs. After excluding these 13 invalid submissions, we have 87 valid submissions from 87 unique workers.

#### 4.1.1. Worker Demographics

Table 1 summarizes workers' demographic information. Of the 87 crowd workers who provided valid submissions, 36 were female (41.38%), while 51 were male (58.62%). Age of participants ranged between 18 and 58 and the majority of them were younger than 35 (87.36%). The majority of the workers (51%) were reported to be unemployed, while from those employed, 73.17% had a full-time job. Most workers had enrolled for or acquired a degree (78.16%), with 51.47% of them pointing to Bachelor's degree. In total, we employed workers from 15 countries, with most workers (77%) currently residing in Portugal (25), United Kingdom (16), Poland (13), and South Africa (13).

**Table 1:** Prolific participant demographics.

	**Variables**	**Statistics**
Age (years)	Range	18–65
	Majority	18–25 (70.11%)
Occupation	Full-time	30
	Part-time	11
	Unemployed	44
	Voluntary work	2
Education	Associate degree	3
	Bachelor's degree	35
	Doctorate degree	1
	High school/HED	16
	Master's degree	12
	Professional degree	1
	Some college, no diploma	13
	Some high school, no diploma	2
	Technical/trade/vocational training	4

#### 4.1.2. Results on Worker Music Sophistication

Table 2 summarizes the results of the GMSI questionnaire on our workers. We contrast our results to results of the original GMSI study (Müllensiefen et al., 2014), which covered a large population sample of participants *n* = 147, 663 that voluntary completed the questionnaire, on BBC's *How Musical Are You?* online test. Participants were mainly UK residents (66.9%) and, in general, from English-speaking countries (USA: 14.2%, Canada: 2.3%, Australia: 1.1%), with 15.9% having non-white background. The sample contained a large spread on education and occupation demographics, where only 1.8% claimed working in the music domain. To some extent, this study is considered representative for the general population in the UK (but is biased toward higher musicality due to the voluntary nature of that study). As such, we can assume a certain disposition and affinity to music from GMSI's population sample, compared to ours where the incentives where monetary.

**Table 2:** GMSI range, median, mean, and standard deviation.

	**Range**	**Median**	**Mean**	**Standard deviation (1σ)**
Active engagement	19–45	31	30.91	5.45
Perceptual abilities	16–45	34	33.62	6.65
Musical training	7–45	17	18.52	9.61
Singing abilities	9–41	28	27.41	6.03
Emotions	18–42	33	33.24	4.28
General music sophistication	40–101	69	69.76	14.20

In our study, the observed General Music Sophistication (μ = 69.76) positions our workers pool at the bottom 28–29% of the general population distribution found in the GMSI study. We observe a similar effect also with the individual subscales with the exception of “Emotions”, for which our workers fare a bit higher (bottom 32–38%).

The result indicates that the self-reported music sophistication of crowd workers is strongly below that of the general population. Most workers had received relatively little formal training in their lifetime. This finding is important for the rest of the analysis, as it indicates *low formal expertise* with music among the crowd workers.

Most workers indicate relatively high perceptual abilities (μ = 33.62, *max* = 45). Here, it is interesting that previous studies (Baharloo et al., 2000) estimate that less than 1% (or 5 people) per 11,000 possess “Absolute Pitch”. In our sample though, 9 workers indicated having this characteristic, little more than the 10% of our sample. This could indicate a possible confusion between quasi-absolute pitch which is related to the familiarity of a person with an instrument's tuning and timber (Reymore and Hansen, 2020), or with relative pitch. Relative pitch is trainable through practice and useful to professional musicians, as they can detect changes in pitch through the relations of tones (5 out of 9 workers who indicated “Absolute Pitch” had scored higher than 30 out of 49 in the “Musical Training” category scale, indicating adequate formal musical training).

Table 3 presents the correlations between GMSI subscales. As the scores of each GMSI subscale follow a normal distribution (Shapiro-Wilk test), we applied Pearson's R test to calculate correlation coefficients. We observe that Perceptual Abilities shows positive correlations with most other subscales (*p* < 0.05), especially with Music Training (*R* = 0.442), Emotions (*R* = 0.380), and Singing Abilities (*R* = 0.463). This finding suggests that the listening skill plays the most important role in crowd workers' music sophistication. We also find significant correlations between Active Engagement and Emotions (*R* = 0.401), and between Singing Abilities and Musical Training (*R* = 0.465). The original GMSI study has shown that different subscales are strongly correlated (*R*>0.486). The difference we observe could be partly explained by the generally lower musical sophistication scores of the crowd workers in our pool.

**Table 3:** Intercorrelations (Pearson's R) of subscales of GMSI scores.

	**Active engagement**	**Perceptual abilities**	**Musical training**	**Emotions**	**Singing abilities**
Active engagement	1.000				
Perceptual abilities	0.262^*^	1.000			
Musical training	0.224^*^	0.442^*^	1.000		
Emotions	0.401^*^	0.380^*^	0.178	1.000	
Singing abilities	0.142	0.463^*^	0.465^*^	0.125	1.000

Statistical significance (p < 0.05) is marked using an asterisk (*).

#### 4.1.3. Results on Objective Music Perception Skills

Mini-PROMS categorizes perception skills as “Basic” if the total obtained score is lower than 18, “Good” if between 18 and 22.5, “Excellent” for values between 23 and 27.5, and “Outstanding” for values over 28 (Zentner and Strauss, 2017). The original Mini-PROMS study covered a total *n* = 150 sample of participants, all recruited from the university of Innsbruck, via email. Most of the participants were students with at least one degree (*n* = 134), aged 27 on average.

We observed (see Table 4) an average of “Good” music perception skills for our workers (μ = 19.53, avg. accuracy 54.25%). Forty-eight out of eighty-seven (55.17%) produced reasonably high accuracy in music skill tests (belonging to “Good” and better categories according to Mini-PROMS results). These figures are lower compared to the results of the original study (Zentner and Strauss, 2017) (μ = 24.56, 68.2% avg. accuracy), a fact that we account to the greater representation of *non-musician* in our workers pool (67.82%), compared to the participants of the original Mini-PROMS study (where only 38.67% identified as non-musicians). However, considering the low formal training amongst the surveyed workers, we consider this result an indication of the existence of useful and somewhat abundant auditory music perception skills among untrained workers. Especially, in the top 10% of workers, ranked according to their total Mini-PROMS values, several achieved quite high accuracy, between 73.6 and 83.3%, which would indicate perception skills between “Excellent” and “Outstanding” in Mini-PROMS's scale. In the following section we will analyse in greater detail the relationship between the measured music sophistication and the perception skills.

**Table 4:** Mini-PROMS range, median, mean, and standard deviation.

	**Range**	**Median**	**Mean**	**Standard deviation (1σ)**
Melody	1.5–9	5	4.98	1.59
Tuning	1–7.5	4	4.22	1.62
Accent	0–9.5	5	5.19	1.84
Tempo	1–8	5	5.14	1.59
Mini-PROMS total	6–30	19.5	19.53	4.98

A similar trend toward lower performance compared to the original Mini-PROMS study can be observed across the other musical aspects: workers correctly identified melody differences with 49.77% avg. accuracy (original study: 64.3%), tuning differences with 52.73% avg. accuracy (original: 68%), accent difference with 51.95% avg. accuracy (original study: 61.5%), and tempo differences with 64.3% avg. accuracy (original study: 81.25%).

The result of the music skill tests is in-line with the result of self-reported music sophistication from GMSI, suggesting that when compared to the populations covered by previous studies, crowd workers generally possess less music perception skills. To deepen the analysis, we calculated the intercorrelation of Mini-PROMS subscales, and made comparison with the original study (Zentner and Strauss, 2017). Since the Mini-PROMS scores across all the subscales follow normal distributions based on the Shapiro-Wilk tests (Hanusz et al., 2016), we carried out Pearson's R tests to get the correlation coefficients and corresponding *p*-values. We find statistical significance on all the intercorrelations. Especially, we find that workers' music skills related to melody are positively correlated with their accent- and tempo-related skills (*R* = 0.551 and *R* = 0.514, respectively), while accent and tempo also shows a moderate correlation (*R* = 0.468). In comparison with the original study, we do not observe large differences in the *R* values, while we did with the GMSI results. The results of the intercorrelation analysis suggests that worker melody, accent, and tempo skills are related with each other in our population too. This is a positive result, that suggests (1) the applicability of this testing tool also on this population, and (2) the possibility of developing more compact tests for music perception skills, for workers' screening or task assignment purposes.

When focusing on the top 10% of workers, we observed an accuracy on “Melody” between 75% and 90%, while the top 5% scored higher than 85%. A person with “Absolute Pitch” would be expected to achieve high accuracy on this test. Only one person in the top 10% had indicated “Absolute Pitch”, but their accuracy was one of the lowest in the group (75%). This could indicate that the person is more likely to not possess such a characteristic. For the subcategory of “Tuning”, the top 10% achieved accuracy between 81.25 and 93.75%, while the top 5% scored higher than 87.5%. On “Accent”, the top 10% reached accuracy between 80 and 95%. Finally, on the subcategory of “Tempo” we measured accuracy of 87.5 and 100% in the top 10%, while the top 5% achieved perfect score of 100%.

These results suggest the presence of a substantial fraction of workers possessing higher music perception skills than expected from their training, although differently distributed. For example, workers who perceived well changes in “Melody”, didn't perform equally well on the other categories. This could indicate that music perception skills do not necessarily “carry over” from one music feature to the other; other workers will be good in perceiving changes in tempo, while others on tuning. This encourages the use of the appropriate set of tests, to identify potentially high performing annotators. Thus, if we take as example beat tracking annotation tasks, it would be more beneficial to focus on testing the rhythm-related perception skills, as the other categories have lower chance to capture the appropriate workers for the task.

#### 4.1.4. Post-task Survey: Equipment and Cognitive Workload

The majority of the workers reported that, during the test, they used headphones (52.87%) (which is very good for musical tasks), earphones (29.54%), and laptop speakers (16.09%) (which are not optimal). All workers reported the quality of their equipment as “Fair” or better quality (55.17% selected “Excellent” and 34.48% “Good”). 96.55% argued that their equipment either does not have any impairment (72.41%) or that the impairment is not annoying (24.13%). Finally, the majority of workers (58.62%) reported near silence conditions, while 31.03% of them reported normal, non-distracting levels of noise. While these conditions are not comparable to lab setups, we consider them to be sufficiently good to accommodate the requirements of our study.

In the NASA-TLX questionnaire, 34.48% of crowd workers reported low “Mental Demand” and 79.31% low “Physical Demand”. “Temporal Demand” was also reported low for the 72.41% of the participants. This low self-reported demand, is reflected also to the majority (55.17%), who reported higher than average “Performance”. Nevertheless, the majority of crowd workers (70.11%) reported average to very high amounts of “Effort” while completing the study, which is not reflected on the perceived mental, physical and temporal demand they experienced. It is also not evident on their “Frustration” levels, since the majority (54.02%) reported low levels.

Using Pearson's R, we found the inter-correlations between the different categories of NASA-TLX. We found high correlation between “Physical Demand” and “Mental Demand”, but also between “Physical Demand” and “Temporal Demand”. Finally, “Frustration” and “Performance” show high correlation between them, which is a reasonable effect.

#### 4.1.5. Identifying Factors Influencing Performance in Mini-PROMS

To better understand factors affecting a participant's performance in Mini-PROMS and therefore their perceptual capabilities on Melody, Tempo, Tuning and Accent, we applied a Multiple Linear Regression, using Ordinary Least Square (OLS) method. We split our analysis based on total score on Mini-PROMS and the individual categories of the test, to study how they are influenced by the rest of the study's categories.

To minimize multi-colinearity between the Independent Variables, we dropped those that showed high correlation between them in our inter-correlation analysis. Analyzing the inter-correlations between all categories, we found similar results to those per part of the study (as analyzed in previous sections). Therefore, NASA-TLX was the only part of the study on Prolific, where high inter-correlation was exhibited between the categories of “Physical Demand” and “Mental Demand”, “Physical Demand” and “Temporal Demand”, “Frustration” and “Performance”. We proceeded to apply OLS, by dropping “Physical Demand” and “Frustration” from the NASA-TLX factors, to decrease colinearity. Correspondingly, for the categorical variables “Occupation” and “Equipment Type”, we only used the “Part Time”, “Voluntary Work”, “Unemployed” and “Headphones”, “Laptop Speakers” for each respective variable.

For the total Mini-PROMS score, we found a significant equation [*F*_(19, 67)_ = 2.948, *p* < 0.000, with *R*^2^ = 0.455], that shows “Perceptual Abilities” and “Musical Training” from GMSI, affect significantly the dependent variable (*p* < 0.05). For each unit increase reported under the “Perceptual Abilities”, a worker showed an increase of 0.2207 point in the total score, while in “Musical Training”, it resulted to a 0.2417 increase.

Running the regression for the “Melody” of Mini-PROMS [*F*_(19, 67)_ = 1.898, *p* = 0.0289, with *R*^2^ = 0.350], we found that their “Occupation” status affected the dependent variable significantly (*p* < 0.05). Their “Part Time” employment seemed to negatively influence their performance in “Melody” test, by −1.2234 points. On the other hand, “Perceptual Abilities” and “Musical Training” from GMSI also affected significantly their performance (*p* < 0.05), increasing it by 0.0827 and 0.0570 points, respectively. Their “Singing Abilities” though, seemed to significantly influence their performance but negatively, where every reported increase on those abilities, resulted to a decrease of −0.0672 point.

The significant regression equation that was found for the “Tuning” category [*F*_(19, 67)_ = 2.301, *p* = 0.006, with *R*^2^ = 0.395] showed that their “Occupation” status was yet again affecting their performance significantly (*p* < 0.05). Those who reported “Unemployed” showed an increase in their performance by 0.9205. Finally, “Musical Training” appears to be another significant factor to their performance in this particular audio test. Each unit increase in the category, resulted in a 0.0709 increase in their performance.

For “Accent”, the regression [*F*_(19, 67)_ = 2.580, *p* = 0.002, with *R*^2^ = 0.422], showed that the “Temporal Demand” the participants experienced, alongside their “Occupation” and “Musical Training”, influenced significantly their performance in this test. An increase in “Temporal Demand” resulted in decrease by −0.0851 point and in “Musical Training”, an increase by a 0.0701 point. “Part Time” occupation is negatively associated with their performance here, leading to a decrease of −1.2801 points.

Finally, for the “Tempo” category of Mini-PROMS, we couldn't find a significant model by applying OLS.

### 4.2. Amazon Mechanical Turk

Of the 100 workers recruited from Amazon Mechanical Turk (MTurk), 9 of them failed at least one attention check question(s); 7 of them provided invalid/none inputs. After excluding these 16 invalid submissions, we have 84 valid submissions from 84 unique workers.

#### 4.2.1. Worker Demographics

We also conducted the same study on Amazon MTurk, in order to see if we can observe similar trends as shown in the last section also on a platform different than Prolific. We gathered 84 crowd workers who provided valid submissions. As seen in Table 5, the age range was between 18 and above 65, while the majority was between 26-35 (52.38%), a relatively older pool compared to the Prolific's one. The majority of them were employed (95.23%), with the 88.75% of them full-time. Most of the participants hold a degree (86.90%), with Bachelor's being the most common (60.27%). Finally, the vast majority of the participants, report the United States of America (89.28%) as their residence, with the rest being spread between Brazil (3), India (3), United Kingdom (1), Netherlands (1), and Italy (1).

**Table 5:** MTurk participant demographics.

	**Variables**	**Statistics**
Age (years)	Range	18–65+
	Majority	26–35 (52.38%)
Occupation	Full-time	71
	Part-time	9
	Unemployed	3
	Retired	1
Education	Associate degree	6
	Bachelor's degree	44
	Doctorate degree	2
	High school/HED	11
	Master's degree	10
	Professional degree	0
	Some college, no diploma	8
	Some high school, no diploma	1
	Technical/trade/vocational training	2

Apart from education, we see a clear difference between the participants from the two platforms on the age, occupation and country of residence categories. In this study, most of the crowd workers from MTurk are older than those on Prolific, employed and residing in USA.

#### 4.2.2. Results on Worker Music Sophistication

In Table 6, we summarize the results of the GMSI questionnaire, regarding the workers on MTurk. As described in Section 4.1.2, we compare the results on this platform, with the results of the original GMSI study (Müllensiefen et al., 2014).

**Table 6:** GMSI range, median, mean, and standard deviation.

	**Range**	**Median**	**Mean**	**Standard deviation (1σ)**
Active engagement	12–46	32	30.57	7.92
Perceptual abilities	18–47	32.5	32.82	5.92
Musical training	7–43	23	21.80	9.40
Singing abilities	9–45	32.5	28.29	8.12
Emotions	7–41	30.5	30.34	5.35
General music sophistication	29–113	75	72.19	18.15

Comparing our collected data to the original GMSI study, we find that the crowd workers of MTurk exhibit a strongly lower overall music sophistication, at the bottom 32% of the original study. They also score low in all sub-categories, with Musical Training being the only category comparing higher to the 37% of the original study's population.

An extremely high number of participants (40.47%), reported having “Absolute Pitch”, which is a highly unlikely portion of the sample, as discussed before. Only 9 of them reported adequate formal musical training, which can indicate a general misconception on the entailing traits of such a phenomenon. The reports are much higher than those on Prolific.

With a quick glance at the values on Table 7, we see that they indicate skewness on the distributions of each category. When running the Shapiro-Wilk normality test (Hanusz et al., 2016), we found that all distributions, except that of “Perceptual Abilities”, are non-normal. For that reason, we used Spearman's ranked test to calculate the correlation coefficients between the GMSI sub-categories.

**Table 7:** Intercorrelations (Spearman's rank) of subscales of GMSI scores.

	**Active engagement**	**Perceptual abilities**	**Musical training**	**Emotions**	**Singing abilities**
Active engagement	1.000				
Perceptual abilities	0.232^*^	1.000			
Musical training	**0.595** ^*^	0.263^*^	1.000		
Emotions	0.213	0.471^*^	-0.052	1.000	
Singing abilities	**0.637** ^*^	0.340^*^	**0.552** ^*^	0.223^*^	1.000

Statistical significance (p < 0.05) is marked using an asterisk (*). Values in bold indicate intercorrelations higher than 0.5.

We find that “Active Engagement”, “Musical Training” and “Singing Abilities” are highly correlated with each other. The positive high correlation between these categories, indicates that crowd workers on MTurk report similarly their aptitude on those GMSI categories. Notably, although not particularly high, there is certainly a positive correlation between self-reported “Perceptual Abilities” and the extent of “Emotions” these crowd workers experience when listening to music (*R* = 0.471).

#### 4.2.3. Results on Objective Music Perception Skills

Table 8 shows the results of MTurk's crowd workers on Mini-PROMS test. The mean overall score shows that the average participant in our sample pool, had lower than “Basic” music perception skills overall (μ = 15.2 42.2% avg. accuracy). This performance is much lower than both the original Mini-PROMS work (Zentner and Strauss, 2017) and the results we retrieved from Prolific. In the Top 10% of the highest performant crowd workers, we see that they score from 61.1% up to 81.94%, scoring from “Good” to “Outstanding”, based on the Mini-PROMS scale.

**Table 8:** Mini-PROMS range, median, mean, and standard deviation.

	**Range**	**Median**	**Mean**	**Standard deviation (1σ)**
Melody	1–8	4.25	4.22	1.56
Tuning	1–7.5	3	3.2	1.33
Accent	0–7.5	4	4.04	1.42
Tempo	1–8	3.5	3.75	1.64
Mini-PROMS	6.5–29.5	14.5	15.2	4.77

Per individual categories, we see that the highest performance that the crowd workers achieved, didn't reach the max of the Mini-PROMS scale of every category except “Tempo”. The avg. accuracy on the “Melody” category, reached 42.2% (original study: 64.3%, Prolific: 49.77%), while on the “Tuning” category, the avg. accuracy was 40% (original study: 68%, Prolific: 52.73%). The participants from MTurk, were able to detect changes on “Accent” features with avg. accuracy of 40.4% (original study: 61.5%, Prolific: 51.95%), while they scored avg. accuracy 46.87% on “Tempo” (original study: 81.25%, Prolific: 64.3%).

Running the Shapiro-Wilk normality test on each Mini-PROMS' category, we find that only the “Melody” one is Gaussian. We used once again Spearman's rank method to calculate the correlation coefficients per category. We found that “Tempo” is highly correlated with “Melody”, while “Tuning” is with “Accent”. These results are not in line with the original PROMS study (Law and Zentner, 2012), but we observe relatively strong correlation between “Tuning”-“Melody” and “Tuning”-“Tempo”, which fall into the PROMS categories of “Sound perception” and “Sensory” skills, respectively.

#### 4.2.4. Post-task Survey: Equipment and Cognitive Workload

The majority of the participants from MTurk used headphones to perform Mini-PROMS (64.28%), while 20.23% used earphones and 15.46% used the speakers of their laptops. Most participants described the condition of their equipment as “Excellent” or “Good”, while one reported it as “Fair”. The majority (66.66%) of the crowd workers, reported any impairment of their equipment as “Impairceptible”, with 15.47% of them describing it as “Perceptible but not annoying”. The rest of the workers reported various degrees of annoying impairments. Finally, 65.47% of the crowd workers performed Mini-PROMS with near silence environmental conditions, while 19.04% reported extreme levels of noise around them. None of the distributions passed the Shapiro-Wilk normality test for each equipment-related category. Running Spearman's rank method, we found no notable correlation between the categories.

In the NASA-TLX questionnaire, 29.76% of crowd workers reported average “Mental Demand”, with 10.71 and 15.67% reporting low or very high mental strain, respectively. 46.43% reported low “Physical Demand” with 36.9% of the total not feeling rushed while performing the study. 27.38% reported average “Performance”, with 22.61% describing their performance as successful. The majority of participants were divided between reporting high effort (27.38%) or moderate difficulty (27.38%). Finally, 34.52% of the crowd workers felt little to no frustration with 20.24% reporting moderate levels.

Using Spearman's rank, we found that “Frustration” is highly correlated with “Physical Demand”, “Temporal Demand” and “Performance”. This shows that the more physical strain and hurried they felt, combined with feelings of failing the task at hand, increased their frustration with the study.

#### 4.2.5. Identifying Factors Influencing Performance in Mini-PROMS

Following the analysis on the results from Prolific, we applied Multiple Linear Regression on the Mini-PROMS categories, using the Ordinary Least Square (OLS) method. For the total Mini-PROMS score as the dependent variable [*F*_(18, 65)_ = 4.742, *p* < 0.000, with *R*^2^ = 0.567], we found that only the “Perceptual Skills” from GMSI and the “Physical Demand” category from NASA-TLX, affect significantly the dependent variable (*p* < 0.05). For each extra point reported under the “Perceptual Skills”, a worker showed an increase of 0.2 points in the total score. On the other hand, a single extra point toward “Very demanding” on the “Physical Demand” category, resulted on a −0.3 decrease of total performance by the worker.

Running the regression for the “Melody” of Mini-PROMS [*F*_(18, 65)_ = 3.443, *p* < 0.000, with *R*^2^ = 0.488], we found that “Physical Demand” category from NASA-TLX affected the dependent variable the most (*p* < 0.05). The effect is negative toward the performance on “Melody”, where each point increase on “Physical Demand” translated to −0.12 point decrease of performance.

The significant regression equation that was found for the “Tuning” category [*F*_(18, 65)_ = 1.849, *p* = 0.0376, with *R*^2^ = 0.339] showed that the most significant factor was yet again the “Physical Demand”. The more physically demanding the study was perceived, it influenced the final score on “Tuning” by −0.09.

For “Accent”, the regression [*F*_(18, 65)_ = 2.130, *p* = 0.0141, with *R*^2^ = 0.371], showed that the “Type” of audio equipment and its “Impairment” affected the workers' performance the most. The “Laptop Speakers” seemed to influenced positively their performance by 1.2193 points, while the less perceptible an “Impairment” was, it was increasing their performance by 0.448 point.

Finally, for the “Tempo” category of Mini-PROMS, we found a significant regression equation [*F*_(18, 65)_ = 4.502, *p* < 0.000, with *R*^2^ = 0.555] that shows that “Physical Demand” and “Perceptual Abilities” influenced the performance on the category most significantly. While an increase in “Physical Demand” decreased the performance by −0.10 point, an increase in the self-reported “Perceptual Abilities” showed an increase of performance on “Tempo” by 0.08.

### 4.3. In Search of Musical Sleepers

Having analyzed each component of our study and using OLS to understand how individual factors could have influenced the workers' performance on the perception skills of Mini-PROMS, we were still interested to investigate how the highly perceptive workers are distributed based on quantifiable expertise. Musical training is an element that can be quantified by questions on credentials, years of education etc, all components that can be retrieved by the respective category in GMSI. It is an attribute that we experts show high proficiency and that a platform could potentially easily store and iterate per worker's profile.

In this study, following the original studies of PROMS (Law and Zentner, 2012) and Mini-PROMS (Zentner and Strauss, 2017), we make the comparisons of levels of Musical Training, against the performance on the categories of Mini-PROMS. Taking a step further, we used as baselines the amount of “Musical Training” that 50% of the original GMSI's population exhibited (27) and the lowest bound of “Excellent” performance (63.98%) on perception skills, as established for Mini-PROMS. We make use of the terms “Musical Sleepers”, to label those who exhibit high performance but reported low training and “Sleeping Musicians”, those who reported extensive training but performed poorly, both terms from Law and Zentner (2012) and Zentner and Strauss (2017).

**Figure 3:**
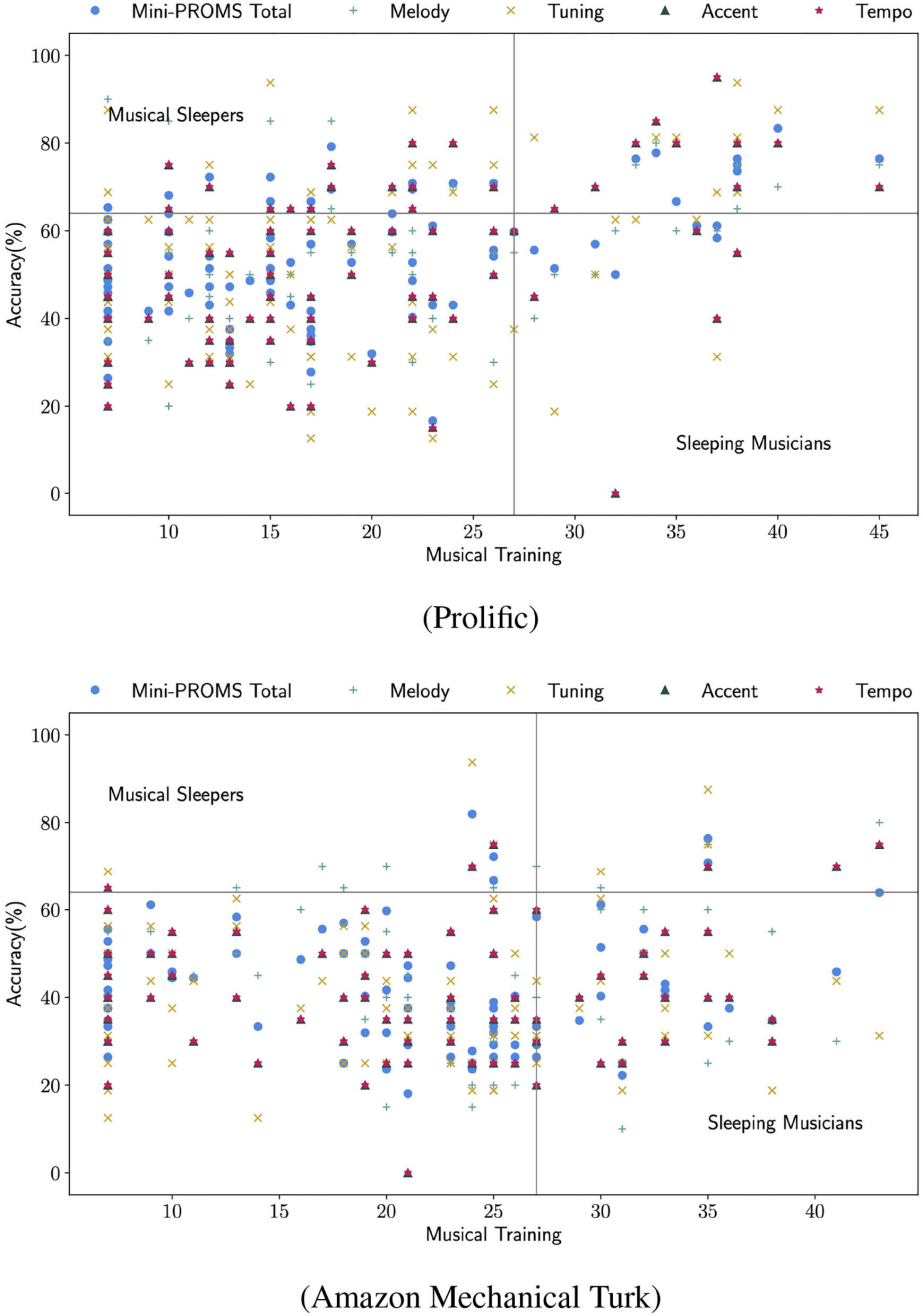
Musical Training (GMSI) and Performance on Mini-PROMS (acc%).

Figure 3 shows a scatter plot per platform, that shows how participants are distributed based on their performance and “Musical Training”. We witness that on both platforms, there is a high number of crowd workers who reported low “Musical Training” [below 50% of original GMSI study in Müllensiefen et al. (2014)] and had relatively low performance in the Mini-PROMS tests. This is to be expected, due to the nature of the domain and the niche skills that are required.

The attention is naturally drawn to the “Musical Sleepers”; a portion of the population that can exhibit relatively high music perception skills, but did not have adequate education. Few people would follow any form of dedicated music studies, making it even more difficult to find them on a crowdsourcing platform. With low expertise being the norm, finding crowd workers with high, untrained, auditory skills among them, is a rare phenomenon that could greatly benefit systems who would make use of such skills. In the case of Prolific, we witness “Musical Sleepers” in a higher number compared to Amazon Mechanical Turk. We cannot draw platform-based conclusions though, since our participant pool was quite small relatively to the actual population of each platform. The presence of these workers is very encouraging, as it shows that it is possible to deploy advanced music analysis tasks on microtask platforms and finding high-value contributors.

In our study, participants from Amazon Mechanical Turk, generally reached lower performance compared to the ones from Prolific. This is an outcome also evident on the high number of “Sleeping Musicians” on MTurk, compared to the smaller portion of the total Prolific participants. These workers reported relatively high musical training, but performed lower than expected from a person of their expertise.

## 5. Discussion

In this study, we extensively measure the musical sophistication and music perception skills of crowd workers on Prolific and Amazon Mechanical Turk. We show that on both platforms, the self-reported music sophistication of crowd workers is below that of the general population and that formally-trained workers are rare. Nevertheless, we found surprisingly refined and diverse music perception skills amongst the top performers per platform. These skills though cannot accurately and easily be predicted by questions.

### 5.1. On Music Perceptual Skills and Predictors

Workers on both platforms exhibited quite diverse set of music perception skills. Among the high performant ones, we found evidence that supports the existence of workers with high accuracy and little to no formal training, namely “Musical Sleepers”, indicating the prospect of high-quality annotations by non-experts on these platforms. Predicting these skills though, can prove far from trivial. To promote reproducibility of our results, we made use of established tools to retrieve domain sophistication (Müllensiefen et al., 2014), perceptual skills (Zentner and Strauss, 2017), perceived workload (Hart and Staveland, 1988), equipment condition (ITU-R BS, 2003) and ambient noise levels (Beach et al., 2012).

In an analysis of workers' reports on other parts of the study, we found per platform, different factors that significantly correlated to their performance. “Musical Training”, a type of expertise that could be thought as a strong indicator of a worker's perceptual skills, showed low significance on the performance of Amazon Mechanical Turk workers. These findings, alongside the high number of “Sleeping Musicians” among the participants from Amazon Mechanical Turk, indicate a notable difference between their reported knowledge and their quantified perceptual skills. On the other hand though, the self-reported “Perceptual Abilities” proved a reliable factor of MTurk workers, as they were significantly related to their performance on Mini-PROMS. This is in contrast to the reported “Perceptual Abilities” of Prolific's workers, which did not significantly correlate to their performance. Aspects of perceived task workload though, as retrieved from NASA-TLX, seemed to significantly correlate on categories of the Mini-PROMS test, on both platforms. Finally, while demographic data appear relevant to aspects of the performance of workers on Prolific, on MTurk equipment showed to play a more important role on the “Accent” test of Mini-PROMS.

The “Active Engagement” category of GMSI, which indicates to what extent a person engages with music as a hobby (frequenting online forums, buying music albums, etc.), did not show any significant correlation to the measured music perception skills of the participants on both platforms. That shows that we cannot reliably use such questions, to infer the skills of the worker; the time/effort spent listening to or discussing about music, can be indifferent of the range of their skills. The same applies to the “Emotions” category, where participants report their emotional response to music. This indicates that music could still evoke emotions to people, even without them perceiving its structural elements.

### 5.2. Implications for Design

***Self-reported Musical Sophistication***.The musical sophistication assessments (GMSI) is a useful tool to evaluate workers' capability in completing music-related tasks. It is however a lengthy questionnaire, which could result in extra cost and worse worker engagement. Reducing the number of question is possible, but with implication in terms of test reliability. For instance, the subscale of Musical Training is positively correlated to their actual music perception skills (and the correlation coefficient is higher than the general GMSI). As music perception skills are of primary relevance when executing music-related tasks, we suggest that in future task design, requesters could consider using the subscale of musical training which only contains 7 items. This could be complemented with novel methods to effectively and precisely predict worker performance to further facilitate task scheduling and assignment.

***Music Perception Skill Assessment***.The Mini-PROMS tool appears to be an effective mean to evaluate worker quality in terms of music skills. Yet, it suffers from the same overhead issues of GMSI. In this case, we suggest to use PROMS or Mini-PROMS as a qualification test, possibly featured by crowdsourcing platforms. Workers could use this test to get the corresponding qualification, to obtain the opportunities to access more tasks, and earn more rewards.

***Music Annotation and Analysis Tasks***.The results of this study indicate that knowledge- and skill-intensive musical tasks could be deployed on microtasks crowdsourcing platforms, with good expectations in terms of availability of skilled workers. However, performance on different skills (Melody, Tuning, Accent, and Tempo) appears to be unevenly distributed. We therefore recommend to analyse the capabilities of the selected crowd and tailor the design of advanced music annotation and analysis tasks to precise music perception skills.

### 5.3. Limitations and Future Work

A main limitation of our study is concerned with the size of the tested population. While we employed workers from two different platforms, our results cannot be generalized per platform. A larger participation pool could potentially aid the generalisability of our findings and lead to more fine-grained insights. Even though our results are based on a population of crowd workers that have received less formal musical training than the average population used in similar studies (Müllensiefen et al., 2014) the use of standardized and validated tests, lend confidence to the reliability of our findings.

Another potential confounding factor in our study is the motivation for participation. We attracted crowd workers using monetary rewards, while in other studies people voluntarily performed their test (e.g., BBC's main Science webpage, Müllensiefen et al., 2014). Such a difference could also explain the differences in observed distributions (musical training and perception skills). However, monetary incentives are a feature of crowdsourcing markets, which makes them appealing in terms of work capacity and likelihood of speedy completion. In that respect, our findings are very encouraging, as they show the availability of both musically educated and/or naturally skilled workers that could take on musically complex tasks.

As demonstrated in our results, workers who perform well in a certain perception category (e.g., “Melody”) do not perform equally well in another (e.g., “Tempo”). In future studies, we encourage the use of perception tests, adjusted and adapted for the specific music task at hand by using the appropriate categories, to accurately select potentially highly performing workers.

In our analysis, we currently made use of Ordinary Least Square Regression to identify factors are associated with the workers' performance on Mini-PROMS. Although this method gave us some first insights, further studies are needed to expand our pool of crowd workers and use other models that can help us find predictors of perceptual skills of workers accurately. This could assist in designing appropriate task assignment methods, to increase the efficiency and effectiveness of crowdsourcing systems that make use of such skills.

In this study, we utilized standardized tools to capture domain-specific characteristics of the workers of a specific platform. Comparing results from their self-reported “connection” to the domain, with those from actively testing their skills, can paint a clear picture of the workers' demographics on a specific domain. While this work is specific to the music domain, we believe that similar workflows can be utilized to study the characteristics of workers on other domains. This holds especially true, as crowdsourcing platforms have diverse user-bases and direct comparisons cannot safely be drawn to studies with highly controlled population samples.

## 6. Conclusion

In this paper, we have presented a study exploring the prevalence and distribution of music perception skills of the general crowd in the open crowdsourcing marketplace of Prolific and Amazon Mechanical Turk. We measured and compared self-reported musical sophistication and active music perception skills of crowd workers by leveraging the established GMSI questionnaire and Mini-PROMS audio-based test, respectively. Our analysis shows that self-reported musical sophistication of crowd workers is generally below that of the general population and the majority of them have not received any form of formal training. We observed differences in the two participant pools, on both their performance and factors which are significantly correlated to it. Nevertheless, we identified the presence of *musical sleepers* on both platforms. Moreover, our analysis shows worker accessibility to adequate equipment. Together, these findings indicate the possibility of further increasing the adoption of crowdsourcing as a viable means to perform complex music-related tasks.

## Data Availability Statement

The raw data supporting the conclusions of this article will be made available by the authors, without undue reservation.

## Ethics Statement

The studies involving human participants were reviewed and approved by Delft University of Technology. The patients/participants provided their written informed consent to participate in this study.

## Author Contributions

IS, SQ, AB, and CL contributed to conception and design of the study. IS implemented the interfaces and online system and wrote the first draft of the manuscript. IS and SQ performed the statistical analysis. All authors wrote sections of the manuscript, contributed to manuscript revision, read, and approved the submitted version.

## Conflict of Interest

The authors declare that the research was conducted in the absence of any commercial or financial relationships that could be construed as a potential conflict of interest.

## Publisher's Note

All claims expressed in this article are solely those of the authors and do not necessarily represent those of their affiliated organizations, or those of the publisher, the editors and the reviewers. Any product that may be evaluated in this article, or claim that may be made by its manufacturer, is not guaranteed or endorsed by the publisher.

## Footnotes

^1^ https://www.prolific.co

^2^ https://https://www.mturk.com

^3^ https://www.last.fm

^4^ https://musicbrainz.org

^5^ https://shiny.gold-msi.org/gmsiconfigurator/

^6^ https://humansystems.arc.nasa.gov/groups/tlx/

^7^ https://vuejs.org

^8^ https://www.limesurvey.org
